# How a major discovery can become a public health failure when used subotptimally: lessons from early nirsevimab implementation

**DOI:** 10.1186/s13052-025-02147-9

**Published:** 2025-11-14

**Authors:** Danilo Buonsenso, Aida Perramon-Malavez, Rosa Morello, Carolina Gentili, Marta Bellorofonte, Antoni Soriano-Arandes

**Affiliations:** 1 Department of Woman and Child Health and Public Health, Fondazione Policlinico Universitario A. Gemelli IRCCS, Rome, Italy; 2 Area Pediatrica, Dipartimento di Scienze della Vita e Sanità Pubblica, Università Cattolica del Sacro Cuore, Largo A. Gemelli 8, Roma, 00168 Italia; 3 Computational Biology and Complex Systems (BIOCOM-SC) group, Department of Physics, Universitat Politècnica de Catalunya (UPC), Catalonia, Spain; 4 Department of Paediatrics, Serveis de Salut Integrats del Baix Empordà, Palamós, Girona, Catalonia Spain; 5 Infection and Immunity in Paediatric Patients, Vall d’Hebron Research Institute, Barcelona, Catalonia Spain

## Abstract

**Background:**

In this Debate, based on our clinical data from the “pre-nirsevimab” and “first year of nirsevimab implementation” bronchiolitis seasons, we challenge the validity of policy decisions that led to partial immunization coverage of eligible newborns and infants during the 2024–25 season in Italy.

**Main body:**

Starting with a pre-nirsevimab prospective cohort of 780 newborns, we documented that 84 (9.2%) were diagnosed with acute bronchiolitis (45 of them (5.8% of the cohort) were RSV positive. 44 patients (5.6%) were hospitalized due to bronchiolitis, of which 7 (0.9%) patients were admitted to the Pediatric Intensive Care Unit. Among hospitalized, 31 infants (70%) had RSV infection. Secondly, we evaluated the impact on bronchiolitis admissions during the first year of nirsevimab use in our region, showing a negligible effect on the most severe cases, probably due to the fact that a low coverage will risk to miss the relatively small number of infants (about 10%) that will develop RSV bronchiolitis in the first year of life. These findings inspired our clinical insights and reflections arguing that without a long-term, cost-conscious approach to implementation, even major scientific breakthroughs like nirsevimab risk becoming public health failures.

**Conclusions:**

Our clinical insights and reflections aim to inspire deeper engagement among policymakers, health agencies, and clinicians to better adapt and integrate RSV preventive strategies—maximizing benefit not only for susceptible infants, but for society at large. In a world of finite healthcare resources, optimizing both the reach and the value of such essential interventions is imperative, given the multitude and diversity of health needs our society is facing.

**Supplementary Information:**

The online version contains supplementary material available at 10.1186/s13052-025-02147-9.

## Background

Bronchiolitis is the most common lower respiratory tract infection in newborns and infants younger than 2 years old. It is the first cause of hospital admission in this age group [1]. Several viruses, such as Rhinovirus, Parainfluenza virus, Metapneumovirus, Influenza virus, Adenovirus, and Respiratory Syncytial Virus (RSV), can cause Bronchiolitis. However, the leading cause of bronchiolitis, especially in children younger than 1 year old, is RSV [2]. According to CDC data, RSV causes approximately 58,000 hospitalizations among children under five years annually [3]. In addition to the acute illness, RSV has potential long-term respiratory complications such as recurrent wheezing and asthma [4].

According to the Italian surveillance network RespiVirNet, RSV caused 49.1% and 22.3% of influenza-like illnesses in children during the 2022–2023 flu season in the < 2 and 2–4 years age groups, respectively [5]. Italian virologic surveillance for the 2023–2024 season identified a total of 2,218 RSV-positive samples in the first 11 weeks of collection, with the majority of cases in patients aged 0–2 years. Additionally, Italian epidemiological studies confirmed a higher RSV incidence and higher need for Neonatal Intensive Care Unit (NICU) for a younger age (≤ 3 months) [6].

Until 2023, Pavilizumab was the only approved prophylaxis against RSV. According to the European Medicines Agency (EMA), it was reserved for children at high risk for RSV disease [7]. A new compound, Nirsevimab, has recently been developed for the prophylaxis of RSV infection to be administered both in prematures or children with risk factors and in healthy term infants. Nirsevimab is a recombinant human immunoglobulin with an extended half-life. It binds the F1 and F2 subunits of RSV fusion protein, blocking viral entry into the host cell [8]. Nirsevimab is a long-acting monoclonal antibody that targets the highly conserved epitope on the RSV F (fusion) protein, preventing viral entry into host cells. In the phase 3 MELODY trial, nirsevimab demonstrated a 74.5% reduction in medically attended RSV-associated lower respiratory tract infections (LRTIs) in healthy infants compared to placebo (95% CI, 49.6 to 87.1; *p* < 0.001) [REF]. In addition, in the MEDLEY trial comparing nirsevimab with palivizumab in preterm infants or those with underlying conditions, nirsevimab showed non-inferior safety and comparable efficacy [REF]. It was approved in Italy in January 2023. It has been demonstrated that a single 50 mg intramuscular dose of Nirsevimab given to newborns and infants in their first year of life provides enough immunity to protect them during the entire season [9].

The advantage of Nirsevimab is that it not only reduces RSV hospitalization rates more than palivizumab, but it also supports a vaccine-like strategy that substantially reduces both treatment costs and direct non-medical costs. A decreased dosage from five injections to one injection indicates that nirsevimab can overcome palivizumab, even without the enhanced potency, from a cost-effectiveness perspective [10].

Several countries, in particular Spanish regions, have implemented a policy toward mass immunization with Nirsevimab of all infants in the first year of life [11]. Others have awaited the season 2024-25 and have either chosen to prioritize most-at-risk patients, or to use the maternal vaccination, which also has been found to be effective in trials [12]. In Italy, after a phase of procedural uncertainty and the absence of national directives, only on 17 October 2024, a deliberation approved Nirsevimab use, having a target population for the first year of 70% of the eligible population [13]. This deliberation provided for the free and voluntary administration of this monoclonal antibody against RSV to infants from 1 November 2024, extending protection also to infants born within the previous 100 days and to children up to two years old with severe diseases, however without providing enough vials to cover all infants under 12 months of age living their first bronchiolitis season [14, 15]. Indeed, eventually region approved the use of nirsevimab born between the end of Kuly 2024 and to November 2024 and those born during the RSV season.

In this debate, we speculate that a strategy targeting only a part of susceptible infants will have a negligible effect on reducing the clinical impact of RSV in young children, but with high costs, paradoxically increasing the costs of national health systems associated directly or indirectly with RSV. In order to evaluate our hypothesis, we used two different but complementary approaches: to understand how many infants of a cohort born soon before the RSV peak in the season will actually develop bronchiolitis without using immunizations, we included a pre-nirsevimab prospective cohort; according to the results of this prospective cohort, we will assess if a partial coverage with Nirsevimab has, indeed, had only a negligible impact on RSV burden in our area, comparing bronchiolitis admissions in the seasons with fours pre-nirsevimab seasons.

## Main text

### What proportion of newborns will develop bronchiolitis?

During the winter 2023-24, when Nirsevimab was already demonstrated as effective and being implemented in other countries but not in Italy, we prospectively followed-up a cohort of term newborns born in our Institution from October 1 st, 2023, to December 31 st, 2023 (representing the most at risk cohort of susceptible young infants during an RSV season). We hypothesized that this would have been the latest “nirsevimab-free” cohort, therefore we followed-up this cohort to know how many of them would develop RSV bronchiolitis and to understand which risk factors are associated with this infection. In fact, at that time, there was already a debate about whether offering Nirsevimab to all infants or not. There were no exclusion criteria for this observation other than prematurity or receipt of palivizumab. No age, gender, or ethnicity restriction has been applied. Details on the methodology of this study are reported in the supplementary material.

### Only a small proportion of newborns will develop bronchiolitis, and only part of them a clinically-relevant disease

During the study period, 780 newborns were enrolled, 391 (50.7%) were male. For more details, see Table 1. During the follow-up period, six patients (0.8%) developed unspecified acute upper respiratory tract infection, and 86 (11.0%) patients developed LRTI. Among LRTI, 2 (0.3%) developed unspecified acute bronchitis, and the rest (*n* = 84) were diagnosed with acute bronchiolitis. Among bronchiolitis, 45 (5.8% of the cohort) were RSV (A and B) positive, the rest (*n* = 40) caused by other viruses. We detected two cases of SARS-CoV-2 infection.

**Table 1:** Shows frequencies of a selection of demographic variables among the analysed cohort

	Total patients (*N* = 780)
Sex	
male	391 (50.1%)
female	389 (49.9%)
Months_old	
Mean (SD)	4.70 (0.918)
Median [Min, Max]	5.00 [3.00, 6.00]
Seasonal_catchup	
catchup	10 (1.3%)
seasonal	770 (98.7%)
Ethnic	
white/caucasian	743 (95.3%)
black/African/Caribbean	23 (2.9%)
hispanic/latino	3 (0.4%)
asian	2 (0.3%)
pacific islander	1 (0.1%)
other/mixed	4 (0.5%)
Missing	4 (0.5%)
Kindergarten	
no	774 (99.2%)
yes	6 (0.8%)
Breastfeeding	
no	134 (17.2%)
yes	644 (82.6%)
Missing	2 (0.3%)
Space_living	
house (with other family members)	776 (99.5%)
shelter center	3 (0.4%)
community space with other families	0 (0%)
other	0 (0%)
Missing	1 (0.1%)
Smokers at home	
yes	209 (26.8%)
no	570 (73.1%)
Missing	1 (0.1%)

Regarding clinical severity, 44 patients (5.6%) were hospitalized due to bronchiolitis, of which 7 (0.9%) patients were admitted to the Pediatric Intensive Care Unit. Among hospitalized, 31 infants (70%) had RSV infection.

These data confirm our hypothesis that less than 10% of eligible newborns will be admitted to hospital due to RSV bronchiolitis during their first RSV season, an observation that will have implications for the implementation of Nirsevimab prophylaxis. If the number needed to treat (NTT) is relatively high (30–57 to prevent one hospitalization in real world studies), it is straightforward to recognize that immunizing only a part (even as high as 50–75%) of RSV eligible infants according to some criteria, such as parents’ acceptance to immunize, or birth period, or availability of the medication in a specific setting, may paradoxically protect either all or none of the eligible infants.

**Fig. 1:**
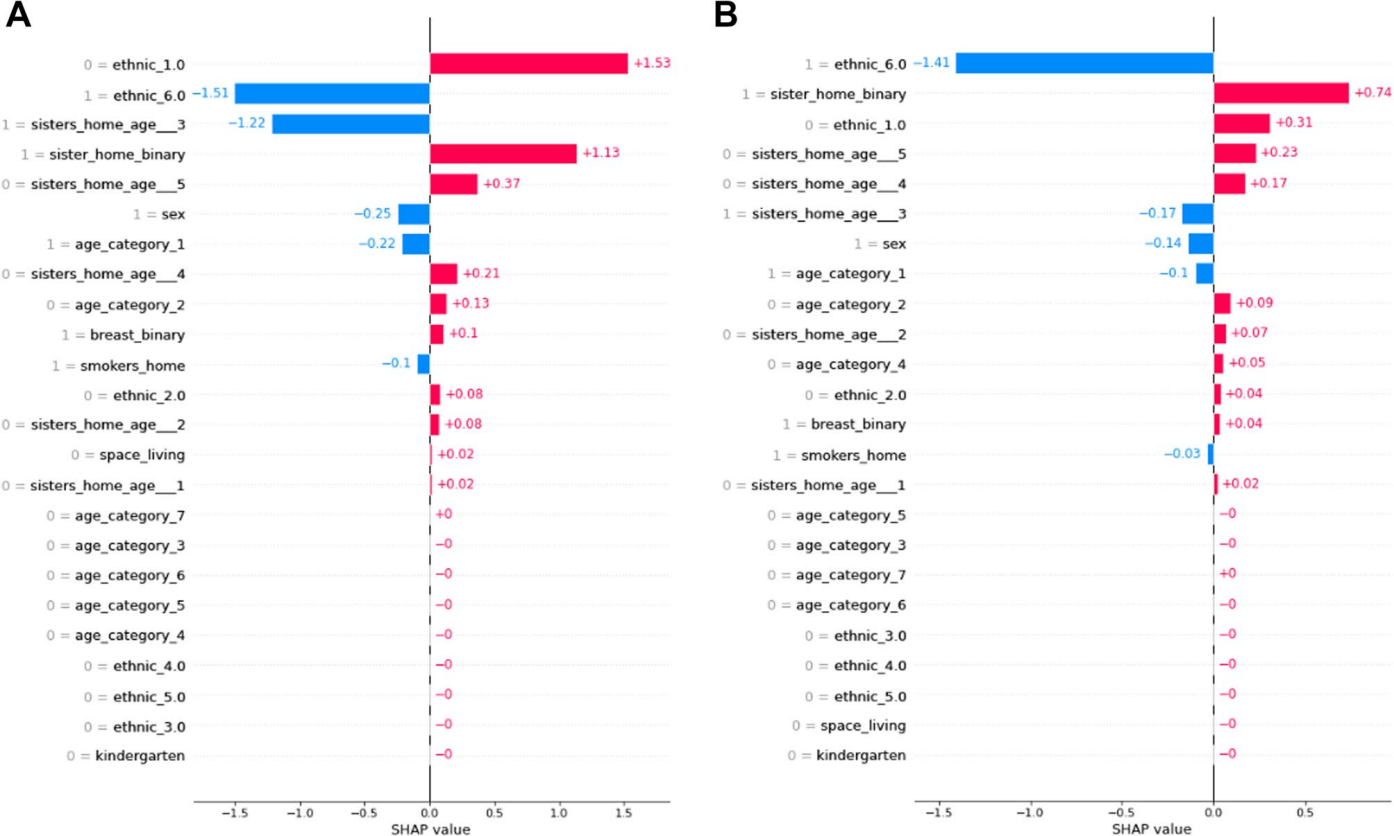
**A** Barplot of SHAP values of the model. Positive values for SHAP indicate the presence of RSV bronchiolitis. Negative values of SHAP indicate the absence of the outcome. A low feature value (blue) indicates the absence of the feature, and a high feature value (pink) indicates the presence of the feature. **B** Barplot of SHAP values of the model. Positive values for SHAP indicate the presence of bronchiolitis, and negative values of SHAP indicate the absence of the outcome. A low feature value (blue) indicates the absence of the feature, and a high feature value (pink) indicates the presence of the feature

Therefore, we aimed to understand which infants have a higher risk of developing bronchiolitis. As shown in Fig. 1A, for RSV bronchiolitis, having young siblings or cousins living in the same household is a predictive factor to be infected by RSV. It also seems that if the sibling or cousin is older than 12 months, the impact on infection is negligible. Besides, sex is also a significant variable since males tend to have a greater probability of infection than females [16, 17]. Instead, as shown in the global model, the age category is not generally important, and ethnicity is not represented diversely enough in this dataset to get any conclusions. Other low-impact conclusions are that not having smokers at home is a bit protective against RSV infection. The same results were found analyzing the all-causes of bronchiolitis (see Fig. 1B).

Our prospective cohort provided us the following information: (i) only a fraction (less than 10%) of susceptible newborns will be admitted to hospital due to RSV bronchiolitis during their first winter, with about 1% of them needing PICU care; and (ii) among them, children with siblings or cousins under 12–24 months of age in the same household or those with smokers within the household are more likely to be infected by RSV developing bronchiolitis; females are less likely to develop RSV bronchiolitis.

Based on these results, a Nirsevimab campaign providing partial and low coverage of eligible newborns would have had a negligible impact on the RSV burden during the season 2024-25, although high economical costs. In Italy, as said, Nirsevimab was approved late in 2024 and it was known that the available doses were not enough to cover all infants under 12 months of age during their first bronchiolitis seasons. To understand if a partial approach was useful, we performed a revision of data of bronchiolitis we are prospectively collecting as part of a larger European study with the aim to compare the “Nirsevimab season – 2024-25” with those of the previous 3 years.

### Nirsevimab had a negligible impact during its first year of implementation in a university hospital in Rome

We collected all diagnoses of bronchiolitis made at our Institution from September to March of each RSV season during the last 4 years (see Table 2).

**Table 2:** Compares frequencies of demographic, Microbiological and clinical data of bronchiolitis in a third-level hospital (Rome) during four seasons

	2021–2022 Season	2022–2023 Season	2023–2024 Season	2024–2025 Season
Bronchiolitis Total Number (September-March)	172	250	241	163
Male (%)	97 (56.7)	142 (56.8)	144 (59.7)	103 (63.1)
Female (%)	75 (43.6)	108 (43.2)	97 (40.2)	60 (36.8)
≤ 12 months (%)	149 (86.6)	234 (93.6)	218 (90.4)	157 (96.3)
Preterm GA < 37 (%)	14 (8.1)	29 (11.6)	25 (10.3)	26 (15.9)
Comorbidities (%)	20 (11.6)	11 (4.4)	11 (4.5)	12 (7.3)
RSV (%)	56 (32.5)	78 (31.2)	65 (26.9)	63 (38.6)
Other virus (%)	41 (23.8)	67 (26.8)	43 (17.8)	51 (31.2)
Total Number of hospitalizations (%)	89 (51.7)	96 (38.4)	86 (35.6)	72 (44.1)
Short Stay Observation Unit (% on admissions)	15 (16.8)	12 (12.5)	15 (17.4)	14 (19.4)
Inward (% on admissions)	60 (67.4)	58 (60.4)	53 (61.6)	46 (63.8)
PICU (% on admission)	14 (15.7)	26 (27.0)	18 (20.9)	12 (16.6)
Respiratory assistance (% on bronchiolitis)	70 (40.6)	90 (36)	83 (34.4)	63 (38)
LFNC (% on RA)	31 (44.2)	33 (36.6)	20 (24.0)	6 (9.5)
HFNC (% on RA)	27 (38.5)	34 (37.7)	45 (54.2)	43 (68.2)
CPAP (% on RA)	11 (15.7)	19 (21.1)	10 (12.0)	10 (15.8)
Mechanical Ventilation (% on RA)	1 (1.4)	4 (4.4)	8 (9.6)	4 (6.3)

*GA* Gestational Age, *RSV* Respiratory Syncytial Virus, *PICU* Pediatric Intensive Care Unit, *LFNC* Low Flow Nasal Cannula, *HFNC* High Flow Nasal Cannula, *CPAP* Continuous Positive Airway Pressure, *RA* Respiratory Assistance

As shown in Table 2, during the last season 2024-25 all data regarding bronchiolitis are similar to the previous seasons. Males are slightly more affected than females. Also, microbiological data show a similar circulation of RSV among infants with RSV causing 38.6% of all bronchiolitis during this season. Analyzing the need for hospitalization, data are mostly in line with those of previous years, including the PICU admissions, similar to other post-covid RSV season. The only difference over the years has been the increased use of HFNC, but this is probably due to the implementation of this respiratory assistance technique over time rather than a change in the clinical picture of the patients. There were no significant differences among cases of mechanical ventilation.

To summarize, data from this season do not show a meaningful difference compared to previous ones, neither regarding virus circulation nor about severity of infections (there has been no reduction in hospital admissions or PICU access or respiratory assistance), suggesting insufficient impact of Nirsevimab in our geographical area characterized by partial and insufficient coverage.

### Why nirsevimab may be not as much effective as initially thought?

There is no doubt that Nirsevimab is highly effective in preventing RSV infection, including severe diseases and deaths. The rationale is behind its biological actions but, even more importantly, several randomized controlled trials and rea-world-evidence have demonstrated, in different settings from Europe to Latin America and North America, that Nirsevimab reduced admissions and deaths for RSV bronchiolitis [18, 19]. Even in Italy, in the only one region in the North (Valle d’Aosta) that adopted a universal prevention program during the 2023–2024 season, a positive impact of the strategy was documented [20].

However, our data support that an inadequate prophylaxis campaign with an insufficient coverage may not be effective for several reasons, hence, to prevent wasting budget the immunization campaign must target universal coverage. As we found in our prospective cohort, and also in line with the placebo cohorts of the Nirsevimab trials [21], despite bronchiolitis is the commonest LRTI of childhood, only a relatively small proportion of infants will develop bronchiolitis. To date, it is impossible to recognize which of the infants will develop bronchiolitis and selectively offer them the prophylaxis. This means that even a Nirsevimab coverage of 75% of the eligible infants may miss part, if not all, those 5–10% that will have a clinically relevant bronchiolitis needing hospitalization during a season. Still, the health of these children that not only will get severe disease but that might develop long-term consequences from such infection are to be attained for, and the healthcare costs of present and future treatment and monitoring should be taken into account.

A recent analysis from the Italian Paediatric Society (SIP) [22] supported this opinion, documenting that the immunoprophylaxis carried out in 2024-25 has been extremely inhomogeneous and fragmented. The result was that not all children have had the same chance to be protected from RSV because the regions have launched very different programs, and not all have had access to the same number of doses. To be specific of our geographical context in Rome, Nirsevimab has been administered to newborns during hospitalization from November 25th to February 28th, whereas children born between August 17th and November 24th could receive the monoclonal antibody in an outpatient setting. Moreover, the prophylaxis strategy applied during the 2024–2025 RSV season has resulted in the exclusion of a group of children (those born before August 17th) who entered the first season of RSV and are therefore also susceptible to infection.

Based on our prospective cohort from the pre-Nirsevimab season (2023-24), it was predictable that a partial Nirsevimab coverage in 2024-25 would have potentially lost a significant proportion of children that would have developed bronchiolitis, as even an 80% coverage would risk missing the 10% or less of children that would really develop clinically relevant bronchiolitis. In addition, the exclusion of infants older than six months also left susceptible a non-negligible number of patients that indeed developed bronchiolitis and need hospitalization, as also previously documented [23]. Indeed, the number of admissions and severe cases we had in 2024-25 was mostly in line with previous years.

Even in terms of costs, the latest RSV season has been in line with previous seasons. Considering a mean cost of €1,500 for RSV bronchiolitis admission and €3,800 euro for respiratory failure [24], the impact has been relevant. The 2024-25 season costed about €161,200 for bronchiolitis only in our Institution, compared with €197,400 in 2023-24, €231,400 in 2022-23, and €179,100 in 2021-22. However, the 2024-25 should take into account the costs associated with nirsevimab. Considering about €35,000 newborns have born in the Lazio region (latest estimate in 2023), and a 82.1% coverage in Italy, we can hypothesize (as actual data are not available) that in our region we spent about 2 to 6 millions of euros for Nirsevimab prophylaxis (considering a 25% or 75% coverage, respectively, and a cost for single vial in Italy of 230 euros). As such, the reductions in admissions and costs made in our hospital, which most probably aligns with the other pediatric hospitals in our region as the epidemiological context is exactly similar, are negligible, making overall the health costs associated with RSV in 2024-25 significantly higher than previous years (as they would include the prophylaxis campaign). A document in Germany estimated that the cost-effectiveness would improve and become favorable including indirect costs associated to RSV (e.g., parents missing work to stay at home or hospital with their sick children) [25]. However, several countries have different maternity leave policies that would allow for household care without additional costs. Moreover, with a partial and ineffective immunization campaign, we are not protecting children from the potential long-term consequences of a bronchiolitis infection, which will also be a logistic and economic burden for the healthcare system in the future.

### So what?

Our data open for concrete scenarios and possible solutions. The first straightforward and obvious conclusion is that protecting a small proportion (50–75%) of susceptible infants with Nirsevimab will keep providing little benefits at enormous costs. This was already clear from the initial trials showing a relatively large number needed to treat (NNT), as also in the placebo groups only about 10% of infants developed bronchiolitis, and is further reinforced by our pre-nirsevimab prospective cohort, and by the burden of RSV in our hospital the 24/25 season compared with previous years.

These observations open to the translation of the historical concept of herd immunity of the vaccination field into the world of the RSV passive immunization. Traditionally, it is considered that having a large proportion of immunized people (herd immunity) against a specific pathogen (e.g., measles) will lower the overall amount of virus able to spread in the whole population. As soon as the percentage of vaccinated people drops of a few percentage points, as happens with measles, the number of new infections will increase exponentially. Although RSV passive immunizations are not expected to lower overall the virus circulation, a similar concept can be applied in terms of clinical impact. To be sure to prevent most if not all cases of bronchiolitis a very high proportion will need to be targeted to make a campaign potentially cost-effective. In this scenario, all of the susceptible children should be targeted. A mid-way approach may only results in loss of resources. In this scenario, it would be better to refute the immunoprophylaxis rather than immunizing only some children, which has several ethical implications due to the proven effectiveness of the universal coverage strategy and shows a lack of long-term planning from public health authorities.

Therefore, the first obvious solution would be to ensure immunoprophylaxis to all susceptible children that enter in the RSV season. Of course, this means having all the necessary doses available at the right time, representing a considerable organizational and economic effort. Several governments have discussed the ideal approach. In Italy, 370,000 children were born in 2024, meaning that an 100% coverage with Nirsevimab would cost €85,100,000 euros with current price per vial of 230 euros [26]. Undoubtedly, the Italian government has not been the only one needing to leverage the potential cost-effective benefits of nirsevimab due to the high prices of the prophylaxis. In a global perspective, these high costs will generate an unequal distribution of child health in the different countries of the world depending on their economic capacities, generating a more exacerbated structural classism already present nowadays, as the different impact of LRTI across the globe shows [27].

Another solution to this issue, less expensive, would be implementing a target immunoprophylaxis, identifying possible risk factors predisposing to bronchiolitis admissions. Nevertheless, this would require a knowledge not yet available. It is known that premature infants and those with comorbidities have a higher risk of bronchiolitis, these cohorts were already targeted with palivizumab and their inclusion in preventive strategies would not be question. Wanting to expand this population, our prospective follow-up during the 2023–2024 season (when Nirsevimab had not yet started) documented that children with siblings or cousins under 12–24 months of age in the same household are more likely to become infected with RSV and develop RSV bronchiolitis and that females are less likely to develop RSV bronchiolitis. Also, having smokers household members was a possible risk factor. However, an approach targeting these risk factors is still limited on overall poorly accurate models and would risk being as much a failure as an incomplete coverate.

As a third addition, some countries may point towards a maternal vaccination strategy. Trials have documented that RSV vaccination is also highly effective in preventing RSV bronchiolitis, particularly within the first three months of life [28]. Theoretically, an open study comparing maternal vaccination and infant immunization as a primary aim has never been performed. Therefore, it is scientifically not possible to define one strategy better than the other. Not by chance, some countries like the UK decided to suggest both strategies including cheaper maternal vaccination [29]. However, maternal vaccination may have caveats, since current trials suggest a possibly shorter protection for infants compared with postnatally immunized newborns, and premature newborns from immunized mother would not be protected by maternal antibodies, as antibodies may have not had time of crossing the placenta. In this case, an at-risk premature infant born from an RSV-immunized mother may need to receive Nirsevimab as well, therefore doubling the costs for protection. In addition, signals on higher rates of late preterm deliveries have been found in trials enrolling in low-to-middle income countries [12].

The fourth scenario, and perhaps the most difficult but beneficial, would be that governments may negotiate more toughly with companies, in light of a series of data emerged from the literature. There are several elements that would put governments in an advantageous position at a negotiating table. While Nirsevimab is clearly effective, its costs are currently extremely high and a coverage for all newborns would be extremely costly with current prices. As it is established that about only 10% of newborns will develop bronchiolitis in the first year of life, it means that 90% will receive the dose with no potential benefits. Secondly, a concrete and pragmatic cost-benefit analysis of “nirsevimab-for-all” implementation strategy is not yet available. As such, governments may speculate that in terms of health economics, the cancellation of the nirsevimab strategy may be paradoxically cheaper than immunizing all infants, particularly in countries like Italy where RSV mortality in the first year of life is close to zero. In this context, while countries with economic capacity would deny the acquisition of nirsevimab, other global regions where it would be substantially more beneficial to prevent child death would not be able to account for it in their public health budgets. Therefore, companies either reduce costs or may lose millions of developed doses. Thirdly, the presence of two effective strategies like maternal vaccination and infant immunization, although one may be slightly better than the other, but with huge differences in terms of costs, represent another element of negotiation. Fourthly, as the most important element to have a clinically relevant reduction in RSV bronchiolitis would be to have a coverage close to 100%, the ideal scenario would be a negotiation where companies significantly reduce the costs of each intervention and countries offer both options to families. As for any medical intervention, some people may prefer maternal vaccination, other neonatal immunization, a few reject both. Having more options may lead to financial competition between companies and lead to lower costs. If both strategies are offered at a reasonable cost, this may improve public acceptance and achieve lower costs for each strategy. A similar scenario, moreover, would facilitate those areas where maternal immunization is easier to be performed or others where post-natal immunization would be preferred, based on local resources and healthcare organizations. Fifthly, we cannot forget how SARS-CoV-2 has been reduced to an almost non-harmful virus with low prevalence thanks to a global vaccination campaign, a public-private collaboration and a solidarity strategy, which might be translated to RSV as well. To aim for that, we need to aim for a global adaptation of such immunization strategies, including all eligible age classes, at ultimately affordable costs both to the Global North and South, which would still be profitable to companies that would expand their target markets. Last, but not least, it is always worth remembering that RSV only causes a fraction of all bronchiolitis cases. This is another element for negotiation, as probably a 30–40% of non-RSV bronchiolitis would still be hospitalized every year, therefore further reducing the cost-effectiveness of the preventing strategies.

## Conclusions

In conclusion, we showed how a partial implementation in the first year of use in our setting may represent a public health failure, as small clinical successes would be obtained at huge costs. Indeed, our prospective cohort showed that bout 10% of susceptible newborns develop bronchiolitis, therefore a strategy targeting even a 70% to 80% target population could miss part of those that would ultimately benefit from the intervention. Our data and views will hopefully inspire further discussions between agencies and policy makers on how to improve the adaptation and sustainability of RSV preventing strategies, so much necessary, in order to provide the maximum benefits not only to RSV susceptible children, but to the local and global societies as well. Indeed, we live in a world with limited resources, and it remains necessary to optimize the costs for each healthcare strategy, given the multitude and diversity of health needs that we are facing worldwide. Despite recent discoveries in RSV prevention, our journey toward an RSV-free era is just at the beginnings and cannot be achieved without a global perspective and further data.

## Supplementary Information

Supplementary Material 1

Supplementary Material 2

Supplementary Material 3

## Data Availability

data available upon request to the corresponding author.

## Abbreviations

RSV: Respiratory syncytial virus
NTT: Number needed to treat
PICU: Pediatric intensive care unit
NICU: Neonatal intensive care unit

## References

[1] Loe MWC, Soenong H, Lee E, Li-Kim-Moy J, Williams PC, Yeo KT, Nirsevimab. Alleviating the burden of RSV morbidity in young children. J Paediatr Child Health. 2024;60(10):489–98. 10.1111/jpc.16643. Epub 2024 Aug 16. PMID: 39150043. 39150043 10.1111/jpc.16643

[2] Manti S, Staiano A, Orfeo L, Midulla F, Marseglia GL, Ghizzi C, et al. Update – 2022 Italian guidelines on the management of bronchiolitis in infants. Ital J Pediatr. 2023;49(1):19. 10.1186/s13052-022-01392-6. 36765418 10.1186/s13052-022-01392-6 PMC9912214

[3] https://www.cdc.gov/rsv/infants-youngchildren/index.html#:~:text=Each%20year%20in%20the%20United,age%2C%20the%20higher%20the%20risk).

[4] Shiroshita A, Gebretsadik T, Wu P, Kubilay NZ, Hartert TV. Association between age of respiratory syncytial virus infection hospitalization and childhood asthma: a systematic review. PLoS ONE. 2024;19:e0296685. 10.1371/journal.pone.0296685. 38349900 10.1371/journal.pone.0296685 PMC10863881

[5] Influnet stagione influenzale 2022/2023. Incidenza delle sindromi simil-influenzali in Italia per fascia di età - Settimane 42/2022–17/2023. https://www.quotidianosanita.it/allegati/allegato1683365649.pdf. Accessed 24 Nov 2023.

[6] Fortunato F, Campanozzi A, Maffei G, Arena F, Carri VD, Rollo T, et al. Respiratory syncytial virus-associated hospitalizations among children: an Italian retrospective observational study. Ital J Pediatr. 2024;50(1):45. 10.1186/s13052-024-01617-w. 38454523 10.1186/s13052-024-01617-w PMC10921699

[7] Gonzales T, Bergamasco A, Cristarella T, Goyer C, Wojdyla M, Oladapo A, Sawicky J, Yee J, Moride Y. Effectiveness and safety of Palivizumab for the prevention of serious lower respiratory tract infection caused by respiratory syncytial virus: A systematic review. Am J Perinatol. 2024;41(01):e1107–15. 10.1055/a-1990-2633. Epub 2022 Nov 30. PMID: 36452969; PMCID: PMC11108679. 36452969 10.1055/a-1990-2633 PMC11108679

[8] Ahani B, Tuffy KM, Aksyuk AA, Wilkins D, Abram ME, Dagan R, et al. Molecular and phenotypic characteristics of RSV infections in infants during two nirsevimab randomized clinical trials. Nat Commun. 2023;14(1):4347. 10.1038/s41467-023-40057-8. 37468530 10.1038/s41467-023-40057-8 PMC10356750

[9] https://www.aifa.gov.it/documents/20142/1805944/DETERMINA_9-2023_BEYFORTUS.pdf

[10] Yu T, Padula WV, Yieh L, Gong CL. Cost-effectiveness of nirsevimab and palivizumab for respiratory syncytial virus prophylaxis in preterm infants 29–34 6/7 weeks’ gestation in the United States. Pediatr Neonatol. 2024;65(2):152–8. 10.1016/j.pedneo.2023.04.015. 37758594 10.1016/j.pedneo.2023.04.015

[11] Soriano-Arandes A, Creus-Costa A, Perramon-Malavez A, Andrés C, Vila J, Gatell A, et al. Early experience on universal prophylaxis in infants against respiratory syncytial virus: facts and expectations. Semin Respir Crit Care Med. 2025. 10.1055/a-2531-0968. 39900111 10.1055/a-2531-0968

[12] Kampmann B, Madhi SA, Munjal I, Simões EAF, Pahud BA, Llapur C, et al. Bivalent prefusion F vaccine in pregnancy to prevent RSV illness in infants. N Engl J Med. 2023;388(16):1451–64. 10.1056/NEJMoa2216480. 37018474 10.1056/NEJMoa2216480

[13] Regione Lazio Registro Ufficiale U. 1020755.13-08-2024.

[14] https://www.statoregioni.it/media/lhnb3llv/p-fs-csr-atto-rep-n-188-17ott2024.pdf

[15] https://pro.campus.sanofi/dam/Portal/Italy/resources/vaccines/La-prima-stagione-di-nirsevimab-in-italia/Pubblicazione---La-prima-stagione-di-nirsevimab-in-Italia.pdf.

[16] Suleiman-Martos N, Caballero-Vázquez A, Gómez-Urquiza JL, Albendín-García L, Romero-Béjar JL, Cañadas-De La Fuente GA. Prevalence and risk factors of respiratory syncytial virus in children under 5 years of age in the WHO European region: a systematic review and meta-analysis. J Pers Med. 2021;11(5):416. 10.3390/jpm11050416. 34063453 10.3390/jpm11050416 PMC8155861

[17] Divarathna MVM, Rafeek RAM, Morel AJ, Aththanayake C, Noordeen F. Epidemiology and risk factors of respiratory syncytial virus associated acute respiratory tract infection in hospitalized children younger than 5 years from Sri Lanka. Front Microbiol. 2023;14:1173842. 10.3389/fmicb.2023.1173842. 37434712 10.3389/fmicb.2023.1173842 PMC10330818

[18] Assad Z, Romain AS, Aupiais C, Shum M, Schrimpf C, Lorrot M, et al. Nirsevimab and hospitalization for RSV bronchiolitis. N Engl J Med. 2024;391(2):144–54. 10.1056/NEJMoa2314885. 38986058 10.1056/NEJMoa2314885

[19] Ares-Gómez S, Mallah N, Santiago-Pérez MI, Pardo-Seco J, Pérez-Martínez O, Otero-Barrós MT, Suárez-Gaiche N, Kramer R, Jin J, Platero-Alonso L, Alvárez-Gil RM, Ces-Ozores OM, Nartallo-Penas V, Mirás-Carballal S, Piñeiro-Sotelo M, Malvar-Pintos A, González-Pérez JM, Rodríguez-Tenreiro-Sánchez C, Rivero-Calle I, Salas A, Durán-Parrondo C, Martinón-Torres F, NIRSE-GAL study group. Effectiveness and impact of universal prophylaxis with nirsevimab in infants against hospitalisation for respiratory syncytial virus in Galicia, Spain: initial results of a population-based longitudinal study. Lancet Infect Dis. 2024;24(8):817–828. 10.1016/S1473-3099(24)00215-9 . Epub 2024 Apr 30. Erratum in: Lancet Infect Dis. 2024;24(7):e419. 10.1016/S1473-3099(24)00355-4. PMID: 38701823. 10.1016/S1473-3099(24)00215-9 38701823

[20] Consolati A, Farinelli M, Serravalle P, Rollandin C, Apprato L, Esposito S, et al. Safety and efficacy of nirsevimab in a universal prevention program of respiratory syncytial virus bronchiolitis in newborns and infants in the first year of life in the Valle d’Aosta Region, Italy, in the 2023–2024 epidemic season. Vaccines. 2024;12(5):549. 10.3390/vaccines12050549. 38793800 10.3390/vaccines12050549 PMC11125727

[21] Hammitt LL, Dagan R, Yuan Y, Baca Cots M, Bosheva M, Madhi SA, et al. Nirsevimab for prevention of RSV in healthy late-preterm and term infants. N Engl J Med. 2022;386(9):837–46. 10.1056/NEJMoa2110275. 35235726 10.1056/NEJMoa2110275

[22] https://sip.it/2025/03/27/vrs-protezione-a-macchia-di-leopardo-e-i-bambini-non-sono-tutti-uguali/.

[23] Camporesi A, Morello R, Pierucci UM, Proli F, Lazzareschi I, Bersani G, et al. 2021/22 and 2022/23 post-pandemic bronchiolitis seasons in two major Italian cities: a prospective study. Children (Basel). 2023;10(6):1081. 10.3390/children10061081. 37371312 10.3390/children10061081 PMC10297298

[24] https://www.salutelazio.it/documents/10182/23074243/Allegato+1/01ffff45-43c4-66e4-dcf7-2c9116912c8d.

[25] Epidemiologisches Bulletin, Aktuelle Daten Und Informationen Zu Infektionskrankheiten Und, Public Health. Stiko. Prophylaxe von RSV-Erkrankungen Mit nirsevimab Bei Neugeborenen und Säugling. Volume 26. Robert Koch Institut; 2024. p. 27. Juni 2024.

[26] https://www.simri.it/wp-content/uploads/2024/12/302.pdf.

[27] Yu X, Wang H, Ma S, Chen W, Sun L, Zou Z. Estimating the global and regional burden of lower respiratory infections attributable to leading pathogens and the protective effectiveness of immunization programs. Int J Infect Dis. 2024;149:107268. Epub 2024 Oct 15. PMID: 39413960. 39413960 10.1016/j.ijid.2024.107268

[28] Kampmann B, Madhi SA, Munjal I, et al. Bivalent prefusion F vaccine in pregnancy to prevent RSV illness in infants. N Engl J Med. 2023;388(16):1451–64. 10.1056/NEJMoa2216480. 37018474 10.1056/NEJMoa2216480

[29] https://www.gov.uk/government/publications/respiratory-syncytial-virus-rsv-vaccination-programmes-letter/introduction-of-new-nhs-vaccination-programmes-against-respiratory-syncytial-virus-rsv

