# Supplementary material for: How a major discovery can become a public health failure when used subotptimally: lessons from early nirsevimab implementation
Source: Ital J Pediatr. 2025 Nov 14;51:301. doi: 10.1186/s13052-025-02147-9 (PMC12619484; doi:10.1186/s13052-025-02147-9)
Supplement: Supplementary file 2 — Supplementary Material 2 [file 13052_2025_2147_MOESM2_ESM.docx]

**Supplementary material**

**Methodology of the prospective cohort**

Data were collected using an electronic case form report (REDCap© web platform). Two pediatricians (one of them the PI, a specialist in pediatric infectious disease), one pediatric trainee, and one medical student composed site staff. We aimed to enroll all the newborns of our hospital during the first post-natal visit. After a consent inform was signed, sociodemographic data for each patient was obtained. Enrolled patients were followed up during RSV season to identify any RSV-associated disease, specifically low respiratory tract infections (LRTI). To this aim, both passive and active surveillance were carried out by site staff. Patients were adequately trained to refer to pediatricians for all respiratory episodes. In case of suspected respiratory infection, a pediatric visit at the investigator site was scheduled. Moreover, at the end of the study period, phone contact was made by site staff to recover any unreported respiratory infection. For each confirmed respiratory episode, clinical and microbiological data were collected. If a patient had more than one episode, data from all episodes have been stored.

For this study, the CRF of each patient has the following sections:

1_ Patient data: This section included demographic data (birthday, gender, and ethnicity) and epidemiological information (kindergarten, breast-feeding, houseparent, smokers at household, other children living at the house such as sister/brother/cousin)

2_Comorbidities and vaccination status

3_Respiratory infectious episode: This section included information about start and end date, clinical signs and symptoms, pulmonary score, radiological, microbiological examination, and severity (need of hospitalization, oxygen and therapy). The section also investigates concomitant or previous respiratory signs and symptoms (during the last week) in parents and/or children living with the patient.

4_ Outcome: The section reported the final diagnosis of respiratory infection (upper or lower respiratory infection, and in the case of lower infection, the type among bronchitis, bronchiolitis, and pneumonia) and outcome (cured, sequelae, or death).

For the study purpose, the variable sex, ethnic, kindergarten (which refers to going or not to the kindergarten), breast_binary (being breastfed or not), space_living (the type of household), smokers_home (the presence of smokers at the household), sister_home_binary (if the patient lives with paediatric age siblings or cousins), sister_home_number (the number of the latter variable), sisters_home_age (divided in 5 categories depending on the age of the sibling/cousin: 1- 10-12m, 2- 12-24m, 3- 24-48m, 4- 48-60m, 5- >60m), final_diagnosis_code and age at recruitment, which we computed as the difference between the date of recruitment and the date of birth, were incorporated. The age was computed in months and the patients were categorized as 1- <1m, 2- 1-2m, 3- 2-3m, 4- 3-4m, 5- 4-5m, 6- 5-6m, and 7- >6m.

Two outcomes were assessed, RSV bronchiolitis and all-causes bronchiolitis, with the aim to generate two datasets, coded as binary (1 if present and 0 if not present).

Both datasets were preprocessed, removing variables having more than 80% of missing values. A consequent missing analysis was performed, searching for the rows with missing values in the remaining variables. When the number of patients was lower than ten, we removed this variable from the analysis. Finally, all variables to binary features were converted, hence ending up with two sets of 768 patients and 25 features.

To determine which of the features are the most predictive of each outcome, two supervised machine learning models (XGBoost and logistic regression) were developed, and their performance was evaluated. The one with the greatest performance was studied using the SHAP methodology in Python. We accounted for the unbalanced nature of our sets (only 43 patients having RSV-related outcome vs more than 700 without infection) we introduced a weighted loss function to the models. To avoid overfitting, we included a stratified 5-fold cross-validation.

In addition, since the model performances this way were close to random guessing (AUC of 0.5), the simplest yet most effective model, logistic regression was chosen, and to compensate the underrepresentation of one class compared to the other, we applied Synthetic Minority Over-sampling Technique (SMOTE) to oversample the minority class. This is a very well-known technique to up-sample the minority classes while avoiding overfitting. We evaluated the performance of our models, computing the precision, recall, and f1 scores and the area under the curve (AUC).

On another hand, to ascertain the results obtained, a principal components analysis was performed, using multiple correspondence analysis (MCA), which is like PCA but for categorical, binary variables. We obtained the two principal components and analyzed which 10 features are more explanatory of them.
