# Supplementary material for: How a major discovery can become a public health failure when used subotptimally: lessons from early nirsevimab implementation
Source: Ital J Pediatr. 2025 Nov 14;51:301. doi: 10.1186/s13052-025-02147-9 (PMC12619484; doi:10.1186/s13052-025-02147-9)
Supplement: Supplementary file 1 — Supplementary Material 1 [file 13052_2025_2147_MOESM1_ESM.docx]

Dear Editor

I am pleased to submit our Personal View entitled **"How a Major Discovery Can Become a Public Health Failure: Lessons from Early Nirsevimab Implementation"** for your consideration in *IJP*.

This manuscript draws from our direct clinical experience during the bronchiolitis seasons before and after the introduction of nirsevimab in Italy, and offers a critical reflection on the early real-world implementation of RSV immunization strategies. While both maternal RSV vaccination and post-natal nirsevimab have demonstrated high biological efficacy in randomized controlled trials, the goal of meaningful public health impact has not yet been fully achieved. Current strategies suffer from limited coverage, high costs, and insufficient long-term planning. Without addressing these challenges, there is a risk that even the most promising biomedical advances may fall short of delivering tangible societal benefit.

We believe it is time to shift the conversation from efficacy to **effective implementation**. Our data and perspective underscore the need for coordinated, resource-sensitive policies that maximize impact. We hope this contribution will help spark deeper dialogue between clinicians, agencies, and policymakers on how to optimize the deployment of RSV-preventive tools—not just to reduce hospitalizations among vulnerable infants, but to ensure equitable and sustainable public health gains.

This manuscript is original, has not been published elsewhere, and is not under consideration by another journal. All authors have approved the final version and declare no competing interests.

Thank you for considering our submission. We would be honored to contribute to the high-level discussion championed by *IJP*.

Sincerely,

Danilo Buonsenso

On behalf of co-authors
