# Supplementary material for: How a major discovery can become a public health failure when used subotptimally: lessons from early nirsevimab implementation
Source: Ital J Pediatr. 2025 Nov 14;51:301. doi: 10.1186/s13052-025-02147-9 (PMC12619484; doi:10.1186/s13052-025-02147-9)
Supplement: Supplementary file 3 — Supplementary Material 3 [file 13052_2025_2147_MOESM3_ESM.docx]

Dear Editor and Reviewer,

thank you for your comments ultimately aimed at improving our paper. Please find below a point-by-point response to your comments. Changes have been highlighted in the marked version of the manuscript.

Reviewer reports:

Abstract
The abstract generally summarizes the paper's scope and message, identifies the context and contrasts clinical efficacy trials with real-world outcomes. However, it reads more like a policy argument than a summary of results. It does not quantify nor briefly mention the authors' empirical findings. Adding a sentence summarizing the main observational result would make the abstract more informative. The abstract is somewhat wordy.

*Thank you very much, we have reformulated the abstract, still keepining in mind that the paper is thoughts as a “different view” paper rather than a traditional original study*

Introduction
It is recommended that the Introduction also include the mechanism of action of nirsevimab and its results in clinical trials (for example, protection rates against lower respiratory tract RSV infections, and direct comparisons with palivizumab), supported by concrete data. The Introduction does not clearly explain Italy's nirsevimab immunization policy.

*Thank you, we have added both sections*

Main Text
The authors should clarify the cohort size, inclusion criteria, and follow-up procedures. The cohort follow-up is mentioned but actual sample size or how "susceptible newborns" are defined is not specified.

*We are sorry, these information are all included in the supplementary material. As this is a debate/personal view paper, we have formatted it in a “special way” and all the details of the methodological approach more tipical of an original paper are included in the supplementary material, otherwise we would lose the debate format.*

It appears they observed <10% hospitalization rate in the cohort, but without a denominator that statistic is hard to interpret.

*We mentioned that during the study period, 780 newborns were enrolled, 391 (50.7%) were male. For more details, see Table 1. So 780 is the deonminator*

The authors should compare their data with data from other countries.

*Data from countries like spain with a high coverage and good effects in prevention are discussed in the discussion: several randomized controlled trials and rea-world-evidence have demonstrated, in different settings from Europe to Latin America and North America, that Nirsevimab reduced admissions and deaths for RSV bronchiolitis. In particular: “There is no doubt that Nirsevimab is highly effective in preventing RSV infection, including severe diseases and deaths. The rationale is behind its biological actions but, even more importantly, several randomized controlled trials and rea-world-evidence have demonstrated, in different settings from Europe to Latin America and North America, that Nirsevimab reduced admissions and deaths for RSV bronchiolitis [18,19]. Even in Italy, in the only one region in the North (Valle d’Aosta) that adopted a universal prevention program during the 2023-2024 season, a positive impact of the strategy was documented [20].”*

The conclusions often generalize beyond the data without presenting statistical analysis or considering alternative explanations.

*Thank you, we have now shortened the discussion and focused more on main data and take home messages*

Overall Assessment
The scientific strength of the article is tempered by its reliance on data from a single hospital site and a relatively small prospective cohort. Throughout the text, replacing subjective or emotive words with precise epidemiologic terms would strengthen the manuscript, narrow data scope, some unsupported generalizations, and overly forceful language in places.

Thank you very much, we cut several sections and toned down the manuscript as you suggested.

Dr Danilo Buonsenso

On behalf of co-authors
